# A Leu to Ile but not Leu to Val change at HIV-1 reverse transcriptase codon 74 in the background of K65R mutation leads to an increased processivity of K65R+L74I enzyme and a replication competent virus

**DOI:** 10.1186/1743-422X-8-33

**Published:** 2011-01-21

**Authors:** HimaBindu Chunduri, David Rimland, Viktoria Nurpeisov, Clyde S Crumpacker, Prem L Sharma

**Affiliations:** 1 Medical Research 151MV, Veterans Affairs Medical Center, 1670 Clairmont Road, Decatur, Georgia 30033, USA; 2 Division of Infectious Diseases, Veterans Affairs Medical Center, and Emory University School of Medicine, 1670 Clairmont Road, Decatur, Georgia 30033, USA; 3 Division of Infectious Diseases, Beth Israel Deaconess Medical Center, Harvard Medical School, 330 Brookline Avenue, Boston, MA 02215, USA; 4 Department of Microbiology and Immunology, Emory University School of Medicine, 1510 Clifton Road, Atlanta Georgia 30322, USA

## Abstract

**Background:**

The major hurdle in the treatment of Human Immunodeficiency virus type 1 (HIV-1) includes the development of drug resistance-associated mutations in the target regions of the virus. Since reverse transcriptase (RT) is essential for HIV-1 replication, several nucleoside analogues have been developed to target RT of the virus. Clinical studies have shown that mutations at RT codon 65 and 74 which are located in β3-β4 linkage group of finger sub-domain of RT are selected during treatment with several RT inhibitors, including didanosine, deoxycytidine, abacavir and tenofovir. Interestingly, the co-selection of K65R and L74V is rare in clinical settings. We have previously shown that K65R and L74V are incompatible and a R→K reversion occurs at codon 65 during replication of the virus. Analysis of the HIV resistance database has revealed that similar to K65R+L74V, the double mutant K65R+L74I is also rare. We sought to compare the impact of L→V *versus *L→I change at codon 74 in the background of K65R mutation, on the replication of doubly mutant viruses.

**Methods:**

Proviral clones containing K65R, L74V, L74I, K65R+L74V and K65R+L74I RT mutations were created in pNL4-3 backbone and viruses were produced in 293T cells. Replication efficiencies of all the viruses were compared in peripheral blood mononuclear (PBM) cells in the absence of selection pressure. Replication capacity (RC) of mutant viruses in relation to wild type was calculated on the basis of antigen p24 production and RT activity, and paired analysis by student t-test was performed among RCs of doubly mutant viruses. Reversion at RT codons 65 and 74 was monitored during replication in PBM cells. In vitro processivity of mutant RTs was measured to analyze the impact of amino acid changes at RT codon 74.

**Results:**

Replication kinetics plot showed that all of the mutant viruses were attenuated as compared to wild type (WT) virus. Although attenuated in comparison to WT virus and single point mutants K65R, L74V and L74I; the double mutant K65R+L74I replicated efficiently in comparison to K65R+L74V mutant. The increased replication capacity of K65R+L74I viruses in comparison to K65R+L74V viruses was significant at multiplicity of infection 0.01 (p = 0.0004). Direct sequencing and sequencing after population cloning showed a more pronounced reversion at codon 65 in viruses containing K65R+L74V mutations in comparison to viruses with K65R+L74I mutations. In vitro processivity assays showed increased processivity of RT containing K65R+L74I in comparison to K65R+L74V RT.

**Conclusions:**

The improved replication kinetics of K65R+L74I virus in comparison to K65R+L74V viruses was due to an increase in the processivity of RT containing K65R+L74I mutations. These observations support the rationale behind structural functional analysis to understand the interactions among unique RT mutations that may emerge during the treatment with specific drug regimens.

## Background

Multidrug resistance (MDR) mutations evolve due to incomplete suppression of viral replication during treatment of HIV-infected patients. The preferential selection and persistence of one mutation relative to another, however, is not well understood. Specifically, the rare combinations of mutations have not been analyzed in depth. As novel nucleoside reverse transcriptase inhibitors (NRTI) continue to evolve and be employed as a component of highly active antiretroviral therapy (HAART), rare combinations and/or new combinations of RT mutations will appear more frequently.

Reverse transcriptase (RT) mutations K65R and L74V/I are selected by several antiretroviral drugs and play important roles in drug susceptibility and/or maintenance of viral load during treatment of HIV-1-infected individuals. Interestingly, prevalence of these mutations in relation to M184V is strikingly low. Analysis of database (Monogram Biosciences, South San Francisco, CA) have shown that thymidine analogue mutations (TAMs) and M184V are the most common (>25%) followed by L74V/I (11%) and K65R (3.3%) mutations during clinical trials [1-3]. Since the prevalence of these mutations have been looked in conjunction with other multidrug-selected mutations, it is not possible to predict the interaction among various mutations and subsequent genotypes.

The selection of K65R and L74V on the same genome is extremely rare. Interesting observation regarding the absence of selection of K65R and L74V in the same virus by Bazmi *et al. *(2000) was revealed during passaging of HIV-1 in the presence of (-)-β-D-dioxolane-guanosine (DXG). This study showed that K65R and L74V were selected during passaging of HIV-1 LAI in the presence of DXG albeit in different viral genome [4]. We subsequently demonstrated that mutations K65R and L74V are mutually exclusive and a R→K reversion occurs at RT codon 65 during replication of virus in peripheral blood mononuclear (PBM) cells in the absence of drugs [5]. These analyses provided the potential mechanism for the extreme rarity of the double mutant in HIV-infected patients. Similar to K65R+L74V, K65R+L74I is also rarely observed in the absence of other mutations [6-8]. Structurally, valine has two methyl groups, whereas isoleucine's branches are one methyl and one ethyl group. Therefore, isoleucine (Ile or I) has an additional methyl group as a side chain in comparison to valine (Val or V). As a consequence Ile has a longer side chain. We hypothesized that L74I in combination with K65R will have a more profound effect on RT resulting in a highly crippled virus. To delineate the differences between valine and isoleucine changes at codon 74 in the background of K65R, we created site directed mutants and performed replication kinetics assays in PBM cells and MT-2 cells, and *in vitro *RT processivity assays. We show here that in contrast to our hypothesis, the L74I change leads to a replication competent virus in the background of K65R. Additionally, virion-associated RT containing K65R+L74I mutations showed increased processivity in a single round of reverse transcription in comparison to K65R+L74V.

## Methods

### Chemicals and medium

Radionucleotides, (*methyl*-^3^H)dTTP and [α-^32^P]dTTP were purchased from Perkin Elmer, (Shelton, CT); poly(rC)-poly(dG)_12-18 _was purchased from Amersham Pharmacia Biotech, (Piscataway, NJ); and Polynucleotide poly(rA) and primer oligo(dT)_12-18 _were purchased from Boehringer Mannheim (IN). The oligonucleotides used for mutagenesis were synthesized and high pressure liquid chromatography purified by Diversified Biopharma Solutions Inc. (Loma Linda, CA). Complete Dulbecco's Modified Eagles Medium (DMEM) containing 10% heat inactivated fetal bovine serum (FBS) and penicillin/streptomycin was used to grow 293T cells. Complete RPMI medium containing 20% FBS, 26 IU of IL-2, penicillin/streptomycin and glutamine was used to culture Peripheral blood mononuclear (PBM) cells. MT-2 cells were grown in RPMI containing 10% FBS, penicillin/streptomycin and glutamine.

### Cells and virus

PBM cells were prepared from Buffy coats received from commercial vendors (Red Cross and LifeSouth Community Blood Center, Atlanta, GA) using Ficoll gradients. Primary human embryonic kidney cells 293T, indicator cell line HeLa-CD4-LTR-β-galactosidase and proviral clone pNL4-3 [9,10] were obtained from the AIDS Research and Reference Reagent Program, Division of AIDS, National Institute of Allergy and Infectious Diseases, National Institute of Health.

### Site-specific mutagenesis and generation of mutant viruses

Various single point mutants were created in the background of proviral clone pNL4-3 by using pALTER^-1 ^mutagenesis system of Promega (Madison, WI) according to manufacturer's guidelines and our previously described protocols [11,12]. Mutagenic oligonucleotide pNL74I 5'-GAAATCTACTATTT TTCTCCAT-3' was used to create L74I mutation in the background of NL4-3 (wild type) and NL4-3 containing K65R mutation. Mutants K65R, L74V, and K65R+L74V that have been previously analyzed for replication capacity and *in vitro *RT processivity were used as controls [12,13]. Viruses were produced using SuperFect^R ^reagent (Quiagen, Valencia, CA) and manufacturer's guidelines. Cells (293T) were split into 60 × 10 mm dishes 24 h-48 h prior to transfection. To generate virus the complex containing 10 μg of DNA in 150 μl of serum-free medium and 30 μl of SuperFect reagent was incubated at room temperature for 10 min. One ml of complete DMEM was added drop by drop onto 293 cells that were washed once with phosphate buffer saline (PBS). Cells were incubated at 37°C in the presence of 5% CO_2 _for 3 h. The remaining medium-complex was removed and the cells were washed with 4 ml of PBS. Four ml of complete DMEM was added and dishes were incubated for 72 h-96 h. Culture supernatants were collected and centrifuged for 5 min at 833g (g = 1.2) to pellet any debris. Culture supernatants were filtered (0.22 μm) and saved in aliquots of 0.5 ml and 1 ml at -80°C. Viral RNA was isolated by QiAamp^® ^viral RNA mini kit (Qiagen Sciences, Valencia, CA). RT PCR was performed using Superscript™ III one-step RT PCR system (Invitrogen, Carlsbad, CA). All the stock viruses were confirmed by sequencing viral RNA using primer 74F, 5'-GTAGGACCTACACCTGTCAAC-3' [14].

### Quantification of virus

Both HIV-1 antigen p24 concentrations as well as RT activity for each stock virus were determined as described previously [12,15]. Briefly, **a**ntigen p24 determination was done according to the manufacturer's protocol using Antigen p24^CA ^ELISA kit (NCI, Frederick, MD). To determine RT activity, one ml of each virus was centrifuged for 2 h at 15,000 rpm in a refrigerated centrifuge [Heraeus Instruments Corp., Model, Biofuge 15R; Rotor, 3743]. Pelleted virions were lysed with 50-100 μl of virus solubilization buffer (0.5% Triton X-100, 50 mM Tris, pH 7.8, 800 mM NaCl, 0.5 mM PMSF, 20% Glycerol), 10 μl of samples in triplicate were mixed with 75 μl of RT assay buffer (60 mM Tris, pH 7.8, 12 mM MgCl2, 6 mM Dithiothreitol, 7 μg dATP) in the presence of 450 ng of poly (rA)-Oligo (dT) and 5 μCi of *methyl*-3H TTP and reactions were incubated at 37°C for 2 h. Entire reaction mixture was overlaid on DE81 filter (Whatman, GE Healthcare). Filters were washed 3 times with 2X SSC buffer, 2 times with absolute alcohol, air dried and the radioactivity was measured in scintillation fluid.

### Determination of viral titer

Viruses produced in 293T cells were quantified in HeLa-CD4-LTR-β-galactosidase cell lines as described elsewhere [10]. Briefly, 20-30% confluent cells in 12-well plate were infected with stock viruses containing 1, 10 and 100 ng antigen p24 in the presence of 20 μg of DEAE-dextran (Pharmacia) per ml. The plates were rocked intermittently every 30 min until 120 min and then 1 ml of DMEM with 10% calf serum was added to each well. After 48 h, the medium was removed and the cells were fixed at room temperature with 2 ml of phosphate-buffered saline (PBS) containing 1% formaldehyde and 0.2% glutaraldehyde for 5 min. The cells were washed four times with PBS and incubated for 50 min at 37°C in 500 μl of a solution of 4 mM potassium ferrocyanide, 4 mM potassium ferricyanide, 2 mM MgCl_2_, and 0.4 mg of X-Gal per ml. The reaction was stopped by decanting the staining solution and washing the cells thrice with PBS. Blue cells were counted at 100X magnification of a light microscope. Infectious units were calculated by counting the number of blue colonies in each dilution and the amount of HIV-1 p24 capsid antigen by ELISA. The amount of virus (antigen p24) required to infect 1 cell was considered equivalent to 1 infectious unit (IU) or multiplicity of infection (MOI) 1.

### Replication kinetics assays

Healthy donor's PBM cells were infected at various MOIs (0.001, 0.01 and 1.0) based upon the IU. Replication kinetics assays were performed by infecting 10 × 10^6 ^PHA-stimulated PBM cells with equivalent amount of viruses. Culture supernatants were collected every other day until day 14 to determine antigen p24, RT activity and genomic RNA sequence. In a parallel experiment 3.0 × 10^6 ^MT-2 cells (0.5 × 10^6^/ml) were infected with 0.001 IU of various viruses and replication kinetics were measured by monitoring RT activity until day 14.

### Quantification of R→K reversion at RT codon 65

We have demonstrated previously that RT containing K65R+L74V is highly unstable and a rapid R→K reversion occurs at RT codon 65 [5]. Homogenous populations of both double mutant viruses, K65R+L74V and K65R+L74I were produced in 293T cells. PHA-stimulated PBM cells (10 × 10^6^) were infected with 0.1 MOI of viruses and reversion of viruses was followed between day 7 and day 28 by sequencing equivalent amount of cDNA products synthesized from viral RNA isolated from culture supernatants at different time points. The relative reversion ratios for double mutants were calculated by comparing the peak heights of nucleotides A/G (AAA/AGA) and T/G (TTA/GTA) at RT codons 65 and 74 respectively. In order to quantify reversion rates, various ratios of wild type cDNA (K65) and mutated K65R cDNA were mixed and sequenced; peak heights were measured for both nucleotides and percentage reversion was calculated according to our previously published protocols [14]. To confirm the ratios of peak heights observed, we performed population cloning in Topo TA cloning vector PCR^R^2.1 (Carlsbad, CA) by cloning RT PCR products and sequencing 20 clones at each time point.

### *In vitro *RT processivity assay

Since various viral (nucleocapsid proteins, integrase) and host factors (p53 and cellular topoisomerase) have been shown to interact with HIV-1 RT [16-23], we compared virion-associated RTs of mutant and wild type viruses in all of our assays. RT processivity assays were performed as described elsewhere [13,24,25]. Briefly, stock viruses supernatants containing 1500 to 3000 ng equivalent of antigen p24 were centrifuged at 16,000 rpm for 2 h at 4°C. RT was dislodged from the pelleted virions by the treatment of 50 μl of 0.5% NP40. The RT activity was determined using homopolymer template/primer [poly rA-oligo d(T)] and α-^32^P dTTP according to published protocols [12,15,25]. Different amount (2 μl, 4 μl, 6 μl) of RT lysates were incubated with 1 μg/ml of poly (rA) and 0.16 μg/ml of oligonucleotide (dT) in the presence of an assay mixture containing 60 mM Tris (pH 7.8), 75 mM KCl, 5 mM MgCl_2_, 0.1% NP40, 1 mM EDTA, and 4 mM DTT at 37°C for 30 min in the absence of radiolabeled dTTP. After the formation of Template-primer-enzyme complex, cDNA synthesis was initiated by the addition of 50 μCi of [α-^32^P] dTTP/ml and 50-fold excess of trap [poly (rC)-oligo (dG)]. The reactions were terminated after 180 min by placing the tubes in ice slurry and addition of the equal volume of buffered phenol. cDNA products were extracted once with phenol:chloroform (25:24) followed by one extraction with chloroform only. In order to normalize the volume of extracted cDNA, equivalent amount of top layer (DNA) was collected after centrifuging the mixture of phenol and DNA solution. The cDNA was precipitated with 2.5 volumes of absolute alcohol in the presence of 2.5 M ammonium acetate. After desalting the precipitated DNA with 70% alcohol, the pellet was vaccume-dried and suspended in 8 μl of sterilized water. Half of the DNA was mixed with formamide-dye mixture and heated at 95°C for two minutes in a water bath. The purified products were run on 6% polyacrylamide sequencing gel electrophoresis at 30 W for 2 h. The wet gels were exposed to autoradiography for 30 min to 2 hr. To determine relative density of bands in the gel, we scanned group of bands using Bio Image Intelligent Quantifier^® ^software (Bio Image Systems, Inc, Jackson, MI).

### Statistical analysis

To compare the replication capacity (RC) of mutant viruses in relation to wild type virus, RC values for 3 independent replication assays were calculated for mutant viruses. A paired analysis with student t-test was performed and p ≤ 0.5 were considered as significant difference. In order to control the variations among sequencing reactions and observed peak heights in chromatograms, we performed regression analysis between observed and expected peak heights for two nucleotides at the same locus [14]. Statistical analysis was conducted to determine the differences in processivity between WT and mutant viruses or among mutant viruses K65R+L74V and K65R+L74I during a single processivity cycle. This analysis was designed to test the hypothesis that for wild type and mutant RTs, cDNA density decreases at the same rate as DNA band number increases. Three to five independent processivity assays were performed for each RT and statistical values that include mean, median, standard deviation and maximum and minimum were obtained. A paired analysis with t-test was performed to compare the density of cDNA products generated by various RTs and p ≤ 0.05 was considered significant difference [12].

## Results

### A Leu→Ile change at RT codon 74 leads to a replication competent virus in the background of K65R (K65R+L74I) in PBM cells

**Figure 1:**
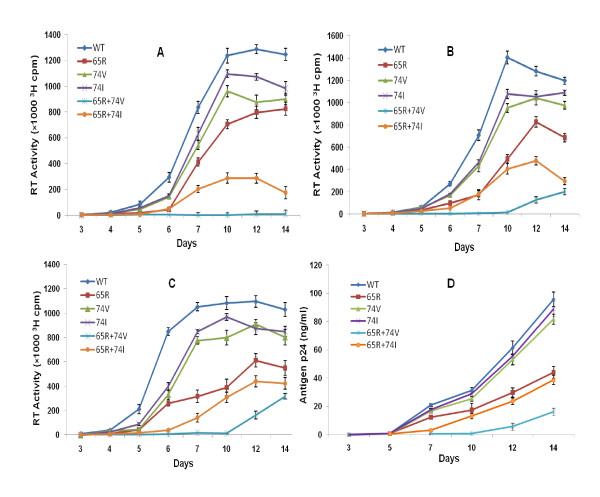
**L→I but not L→V change at RT codon 74 results in a replication competent virus in the background of K65R mutation**. PHA-stimulated PBM cells (10 × 10^6^) were infected with 293T-derived viruses containing MOIs: 0.001 (A), 0.01 (B), 0.1 (C) and 0.01(D) and culture supernatants were collected at various time points. RT activity (A, B, and C) and antigen p24 (D) was determined to monitor viral replication. The plot shows efficient replication with a sigmoid growth curve for K65R+L74I virus suggesting the yield of a replication competent virus. Viruses with K65R+L74V mutant virus did not show measurable RT activity until day 14 at 0.001 MOI. At higher MOIs (0.01 and 0.1), measurable RT activity (B and C) or antigen p24 (D) was observed after day 10 in viruses with K65R+L74V mutation.

We have previously demonstrated that L→V substitution at RT codon 74 in the background of K65R results in a highly attenuated virus [5]. We compared the impact of L→I change on viral replication. Replication capacity (RC) of mutant viruses with respect to WT virus were determined based upon the RT activity (Figures 1A, B, C) or antigen p24 (Figure 1D) values. The pattern of growth curve (sigmoid) obtained with K65R+L74I viruses was similar to WT and point mutants in PBM cells. In contrast to this K65R+L74V viruses showed a longer lag period and initiation of replication resulted in R→K reversion as shown previously (5) (Figures 1A, 1B, 1C and 1D). The replication kinetics pattern in Figures 1B, 1C and 1D indicate a longer lag period of 10 days for the viruses with K65R+L74V mutations when infections were done at 0.01 and 0.1 MOIs. In contrast, K65R+L74I viruses show a lag period of 5 days similar to WT and point mutants K65R, L74V and L74I. At low MOI of 0.001, no measurable growth (RT activity) of K65R+L74V viruses was noted until day 14 (Figure 1A). Since the initiation of viral replication for K65R+L74V virus was observed after day 10, we compared RCs of two double mutant viruses on day 12. Based upon the RT activity (Figures 1A, 1B and 1C), the relative replication capacities of double mutants with respect to WT virus on day 12 in three independent assays were: K65R+L74V [MOI 0.01, RC (0.10, 0.13, 0.11); MOI 0.1, RC (0.14, 0.16, 0.15), and K65R+L74I (MOI 0.01, RC (0.37, 0.42, 0.39); MOI 0.1, RC (0.40, 0.47, 0.44)]. To exclude the possibility of altered RT activity in the measurement of relative RC values of mutant viruses, we also calculated RCs using antigen p24 values (Figure. 1D). The RCs for K65R+L74V and K65R+L74I viruses were 0.09 and 0.38 respectively based upon antigen p24 values of day 12 (Figure 1D). The paired analysis by student *t-test *showed a significant increase (p = 0.0004) in RC of K65R+L74I viruses in comparison to K65R+L74V viruses. These results demonstrated that the L→I change at RT codon 74 improves the replication capacity of the K65R+L74I virus. Based upon the RT activity (Figures 1A, 1B and 1C) the replication capacity of point mutants in three different MOIs (0.001, 0.01, 0.1) were: K65R (0.66, 0.57, 0.53), L74V (0.72, 0.81, 0.78), and L74I (0.79, 0.91, 0.82). Similarly, RCs based on antigen p24 amount (Figure 1D) were: K65R (0.48), L74V (0.86), and L74I (0.90). The relative RCs were: WT > L74I > L74V > K65R > K65R + L74I > K65R + L74V. Based upon the relative growth kinetics demonstrated in the graphs (Figures 1A, 1B, 1C and 1D) we didn't observe any significant differences between RCs calculated by antigen p24 or RT activity determinations. The observed attenuated phenotype of viruses containing point mutations K65R and L74V was in agreement with previous documentations [7,12,15]. We observed slight increase in the RCs of L74I viruses as compared to L74V viruses in different assays but no statistical significance was noted. Previous studies analyzing the risks and incidence of K65R and L74V mutations in the largest single clinic cohort in Europe (The Chelsea and Westminster HIV cohort) have demonstrated that the risk of developing L74V or K65R mutation during HAART was 4.5 and 2.8 cases per 100 person/year, respectively [26]. The decreased frequency of selection of K65R and L74V and the rare occurrence of K65R+L74V on the same HIV genome [6,27] may be related to the observed attenuation of the virus in the presence of these mutations [5,7,12,15].

### Comparison of replication kinetics of mutant viruses in MT-2 cells

**Figure 2:**
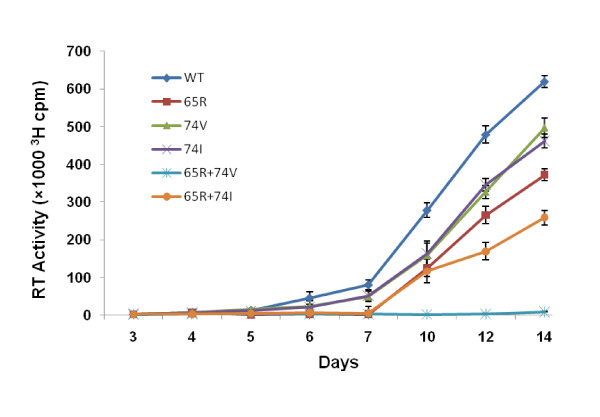
**Efficient replication of viruses containing K65R+L74I mutations in MT-2 cells**. In order to understand the replication of mutant viruses in cells containing higher dNTP pools, 3 × 10^6 ^MT-2 cells (0.5 × 10^6^/ml) were infected with 0.001 MOI of 293 cells-derived viruses. Culture supernatants were collected at various time points and RT activity was determined. The graph shows a more profound difference in replication kinetics of K65R+L74I versus K65R+L74V viruses in MT-2 cells in comparison to that observed in PBM cells.

Since the presence of higher dNTP pools in cells has been shown to influence viral replication capacity and *in vitro *processivity of mutant enzymes [28-32], we performed replication kinetics assays by infecting MT-2 cells that contain inherently higher concentrations of natural dNTPs in comparison to primary PBM cells. Comparison of replication kinetics plot revealed that the L→I but not L→V change at RT codon 74 in the background of K65R results in a replication competent virus. No measurable RT activity was obtained until day 14 for the viruses with K65R+L74V mutations. Control viruses with point mutations, K65R and L74V replicated inefficiently compared to wild type virus, as shown previously [12,15,31,32] but replicated better than the double mutant K65R+L74I (Figure 2). Viruses with L74I mutation replicated similar to L74V viruses.

### Comparison of R→K reversion dynamics at codon 65 for doubly mutant K65R+L74V and K65R+L74I

**Figure 3:**
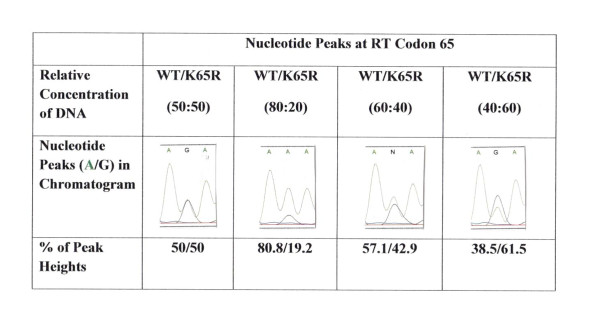
**Correlation between cDNA concentrations and peak heights at codon 65 in chromatogram**. Different ratios of cDNA were mixed and sequencing was performed. Peak heights of wild type 'A' nucleotide and mutated 'G' nucleotide were measured and percentage of both nucleotides was calculated. A strong correlation between cDNA concentration and observed peak heights was obtained in our assay system. The difference between actual peak heights and expected peak heights in relation to DNA concentration was within a range of 2% ( ± 1-2%).

**Figure 4:**
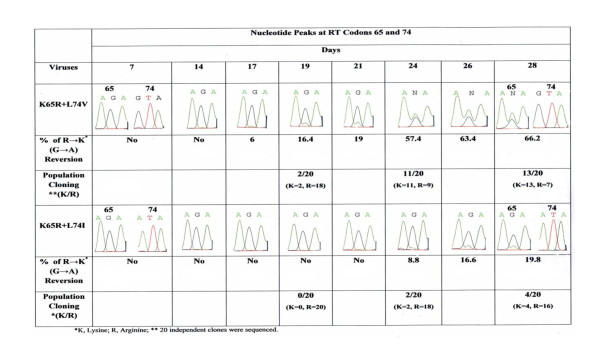
**Comparison of R→K reversion dynamics at RT codon 65 for K65R+L74V and K65R+L74I viruses by direct and population sequencing**. PHA-stimulated PBM cells were infected with equivalent amount (0.01 IU) of the 293 cells-derived doubly mutant viruses. Infectious viruses were sequenced at each time point shown and % R→K reversion was calculated. Population cloning of RT PCR product was performed and 20 independent clones for days 19, 24 and 28 were sequenced. A significant decrease in the reversion was observed with K65R+L74I viruses in comparison to K65R+L74V viruses. No reversion was observed at codon 74 in both double mutant viruses. Reversion data shows that the RT containing isoleucine change at RT codon 74 is much more stable than that with valine change in the background of K65R mutation.

In order to assess the reversion rate among double mutants we sequenced infectious viral RNA at several time points of replication and analyzed peak heights ratios in relation to DNA concentration. To control any variation between different sequencing reactions, we included mixtures of known amount of wild type and mutated (AAA/AGA) cDNA, and generated regression line between ratios of peak heights for 'A' and 'G' nucleotides (A/G) and cDNA concentrations (Figure 3). The percentages of observed and actual peak heights were similar ( ± 2%). These observations were in agreement with our previous documentation [14]. As shown in chromatogram (Figure 4), at RT codon 65 a significant increased R→K reversion was observed for K65R+L74V virus in comparison to K65R+L74I viruses. Comparing extent of R→K reversion on day 28 revealed a 19.8% and 66.2% reversion for K65R+L74I and K65R+L74V viruses respectively. Figure 4 shows that the reversion dynamics for K65R+L74I is clearly different than K65R+L74V viruses. It should be emphasized, however, that K65R+L74V is a non-viable virus and R→K reversion is related to the initiation of replication, suggesting this RT prefers natural dNTP 'A' (AAA, Lys) over 'G' (AGA, Arg) nucleotide for the survival of the virus. In contrast, K65R+L74I virus appears to be replication competent (Figure 1) and no visible reversion at RT codon 65 was observed until day 24 (8.8% reversion). These results suggest that L74I change in the background of K65R leads to an RT which is much more stable as compared to RT with the K65R+L74V mutations. In order to validate the reversion observed in sequence-chromatograms, we performed population cloning of the RT PCR products containing mixtures of parental and revertant viruses. Since visible reversion in sequence-chromatogram of K65R+L74V virus was observed on day 19, we performed population cloning for RNA isolated on days 19, 24 and 28 for both mutants. The sequence analysis of 20 independent clones at each time point revealed that the rate of reversion was significantly high for K65R+L74V viruses in comparison to K65R+L74I viruses. The population cloning results were in agreement with the rate of reversion calculated on the basis of the peak heights of two viruses (Figure 4). No reversion at codon 74 was seen in any of our assays. The rapid reversion of K65R+L74V viruses is also in agreement with the observation that K65R+L74V virus has a longer lag period and abrupt initiation of replication coincides with the detection of R→K revertants in PBM cells during replication of the virus [5].

### Increased *in vitro *processivity of K65R+L74I RT in comparison to K65R+L74V RT

**Figure 5:**
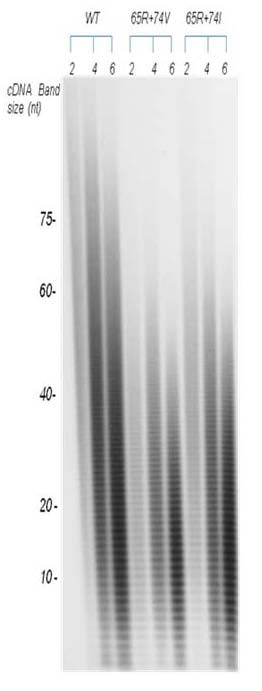
**Demonstration of increased processivity of RTs containing K65R+L74I**. Various mutant RTs were incubated with template/primer poly (rA)-oligo (dT) in the presence of 50 molar excess of trap poly (rC)-oligo (dG) and α-^32^p TTP. cDNA were purified by phenol/chloroform extraction and run on a 6% polyacrylamide gel electrophoresis. Wet gels were exposed to autoradiography. cDNA fragments of different lengths and intensities are shown here. In actual autoradiograph, we were able to observe the largest cDNA bands of 72 nt, 48 nt and 54 nt in length for WT, K65R+L74V, and K65R+L74I respectively. The autoradiograph shows increased intensities of cDNA bands (13-36) synthesized with 4 and 6 μl of RT lysates of K65R+L74I viruses in comparison to K65R+L74V RT lysates (see Figure 6).

**Figure 6:**
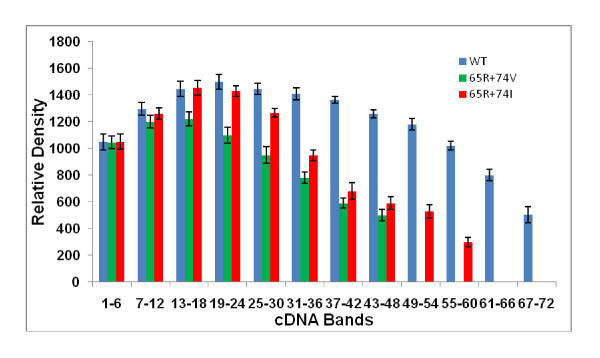
**Quantification of cDNA bands synthesized by WT, K65R+L74V and K65R+L74I RTs**. Groups of 6 bands from bottom to top of each lane were scanned and quantified by Intelligent Quantifier software (Bio Image Systems, Inc., Jackson, MI). The graph shows the cDNA density of bands obtained with 6 μl of RT lysates. RT containing K65R+L74I mutation showed a significant increase in the density of cDNA bands (13-36) in comparison to K65R+L74V RT.

Previous studies have shown the relationship between replication attenuation and in vitro RT processivity of several nucleoside analogue-selected mutants [12,24,25,28,32-34]. We have recently shown that RT with K65R+L74V mutation has a significant decrease in *in vitro *RT processivity as compared to WT and RTs containing point mutations K65R and L74V [13]. To delineate the mechanisms involved in improved replication kinetics of K65R+L74I viruses in comparison to K65R+L74V viruses, we analyzed processivity of virion-associated RTs containing K65R+L74V, and K65R+L74I mutations in *in vitro *processivity assays. RT lysates were prepared by centrifuging culture supernatants containing equivalent antigen p24 concentrations. To determine a single cycle processivity, 2, 4 and 6 μl of RT lysates were incubated with homopolymer poly A and oligo dT (see materials and methods) in the presence of 50-fold excess of poly (rC)-oligo (dG). Purified cDNA products were run on 6% polyacrylamide gel and wet gel was exposed to autoradiograph (Figure 5). We compared the length of the largest fragment obtained during single cycle of processive cDNA synthesis by three RTs. The largest cDNA band for WT, K65R+L74V and K65R+L74I viruses were 66 ± 6, 48 ± 6, 54 ± 8 respectively. We also compared densities of cDNA in a group of 6 bands from bottom to the top of each lane using Bio Image Intelligent Quantifier^® ^software (Jackson, MI). The densities obtained from 3-4 independent assays were averaged and compared with wild type RT and among mutant RTs (Figure 5, Figure 6, Table 1). As reverse transcription reactions with 6 μl of lysates resulted in most prominent cDNA band density with all three RTs (WT, K65R+L74V, K65R+L74I), we calculated statistical differences from lanes designated 6 in Figure 5. We compared significance among cDNA density of WT and double mutant viruses at three locations in the lane. We found no significant difference among densities of bottom six (1-6) bands between WT and K65R+L74V RTs (p = 0.38) and WT and K65R+L74I (p = 0.49) by paired student t-test analysis. However, a significant increase in the densities of bands 25-30 was observed for WT RT in comparison to RTs of double mutants (WT/K65R+L74V, p = 0.001; WT/K65R+L74I, p = 0.01). As largest cDNA band was 48 ± 6 nt for K65R+L74V RTs, we compared the densities for the largest group of bands (43-48) for all three RTs. Clearly, WT RT synthesized increased cDNA molecules resulting in a significant increase in densities of this group of bands (43-48) in comparison to K65R+L74V (p = 0.00007) and K65R+L74I (p = 0.0001). We also performed paired student t-test analysis to determine increased density of cDNA bands synthesized by K65R+L74I RT in comparison to K65R+L74V RT. No significant difference was obtained for shorter cDNA bands 1-6 (p = 0.384) and bands 7-12 (p = 0.237). However significant increase in the densities for larger bands (13-36) synthesized with K65R+L74I RT was obtained. The p values were 0.016 (bands 13-18), 0.007 (bands 19-24), 0.010 (bands 25-30) and 0.023 (bands 31-36) (Figure 5 and Figure 6). Thus, L→I change at RT codon 74 resulted in an increased processivity of RT with K65R mutation. Our analyses of comparative replication kinetics and *in vitro *processivity demonstrated that the improved replication capacity of K65R+L74I virus was due to an increase in the processivity of RT containing K65R+L74I mutant. In summary, K65R+L74I virus showed a shorter lag period (similar to WT and point mutants), increased RC and increased RT processivity in comparison to K65R+L74V viruses, suggesting a different structural constraint on RT with L→I change.

**Table 1:** CDNA density obtained for Wild type, K65R+L74V and K65R+L74I RTs

Group of cDNA Bands^a^	cDNA Density			
		
	WT	65R+74V	65R+74I	p-values
1-6	1153 ± 103.0	1125 ± 79.0	1152 ± 102.0	0.38^b^, 0.49^c^
7-12	1375 ± 80.5	1282 ± 82.5	1332 ± 72.5	0.237^d^
13-18	1545 ± 100.0	1309 ± 89.0	1549 ± 95.5	0.016^d^
19-24	1592 ± 94.5	1202 ± 102.5	1497 ± 70.5	0.007^d^
25-30	1521 ± 76.5	1059 ± 109.0	1321 ± 56.5	0.010^d^
31-36	1483 ± 76.5	854 ± 74.0	1018 ± 68.5	0.023^d^
37-42	1410 ± 46.0	655 ± 65.0	789 ± 109.5	
43-48	1314 ± 55.0	572 ± 72.5	672 ± 82.0	0.00007^e^,.0001^f^
49-54	1254 ± 74.0		614 ± 84.0	
55-60	962 ± 57.5		362 ± 62.5	
61-66	727 ± 73.0			
67-72	606 ± 102.0			

^a ^Groups of cDNA bands from 6 μl lanes of three RTs shown above (Figure 5)

^b ^WT/K65R+L74V and WT/K65R+L74I, identical p values were obtained comparing both double mutants with WT

^c ^WT/K65R+L74I

^d ^K65R+L74V/K65R+L74I

^e ^WT/K65R+L74V

^f ^WT/K65R+L74I

## Discussion

Certain combinations of RT mutations are rare in the clinic and it is conceivable that a specific combination will never be observed due to severe structural-functional constraints on RT which do not allow a viable virus. We have shown previously that K65R and L74V mutations are incompatible and a 65R→K reversion occurs during the replication of double mutant virus K65R+L74V [5]. Biochemical analysis revealed that doubly mutant RT has a significant decreased ability to incorporate natural dNTPs in comparison to wild type RT and K65R RT [29]. Also, virion-associated RT containing these two mutations had a significant decrease in RT processivity in comparison to WT, K65R and L74V RTs [13]. Recent careful screening of an HIV-1 database has revealed the importance of a less studied L→I mutation at codon 74. Similar to L74V, the selection of L74I is also rare in the same HIV-1 genome that contains K65R mutation [1,6,8]. Since 74I possesses an additional side chain as a methyl group in comparison to 74V, we expected a more pronounced processivity defect with the RTs containing both mutations K65R+L74I in the same genome. In contrast, we show here that the K65R+L74I viruses replicated much more efficiently in PBM cells than those containing K65R+L74V. In fact in MT-2 cells, viruses containing K65R+L74I mutations showed a better replication capacity, suggesting the role of higher dNTP concentrations of MT-2 cells in conferring an increased replication of mutant viruses. In parallel to improved replication capacity of K65R+L74I viruses, our reversion assays showed a significant decrease in R→K reversion at codon 65 in K65R+L74I viruses in comparison to those containing K65R+L74V mutation (Figure 4). We speculate that a decreased R→K reversion in K65R+L74I viruses is due to a decreased survival pressure as compared to the viruses with lethal combination K65R+L74V.

In conjunction with improved replication kinetics of K65R+L74I viruses, RT containing K65R+L74I showed a significant increase in *in vitro *processivity in comparison to K65R+L74V RT. Evidently, the side chain of isoleucine improved the processivity of K65R+L74I RT during incorporation of 'T' nucleotide (α-^32^P TTP) rather than imparting a more severe structure-function constraint compared to K65R+L74V RT. Previous mutagenic study of RT codon 74 demonstrated that apart from L74M, other changes L74A, L74G, L74D did not yield enough RT to yield a viable virus [35]. These studies emphasized the effect of severe structure-function constraint of side chains of amino acids at RT codon 74. In contrast, our analysis show that L→I change at RT codon 74 improves RCs of viruses in the background of K65R, suggesting that the specific interaction among amino acid residues at RT codon 65 and 74 could have a different structural constraint. A recent study comparing binary structures of WT and M184I RTs showed that Ile mutation at position 184 with a longer and more rigid beta-branched side chain possibly deforms the shape of the dNTP binding pocket which can restrict dNTP binding resulting in inefficient DNA synthesis at low dNTP concentrations [36].

RT codons 65 and 74 are parts of the highly flexible β3-β4 linkage group in the finger subdomain of the 66 kDa subunit of HIV RT [37]. Analysis of HIV-1 RT crystal structure by Huang et al. (1998) showed that Lys65 and Arg72, main-chain-NH groups of residues 113 and 114 along with two Mg+ ions are involved in coordinating the incoming triphosphate. In the process, Arg72 donates hydrogen bonds to the α-phosphate and the ε-amino group of Lys65 donates hydrogen bonds to the γ-phosphate. These events lead to the finger closure and trapping of the template strand due to the interaction of L74 with the dNTP and template base [37]. Our data suggest that the side chain (methyl group) in isoleucine (74I) conferred a decreased structural constraint on RT to improve the replication of viruses containing K65R+L74I mutations. In contrast to this the major influence observed with K65R+L74V RT may be during reinitiation and not during processive synthesis [5,37].

The effect of compensatory mutations on viral replication and RT has been previously analyzed by several laboratories [34,38,39]. In an era of combination therapy and the selection of MDR mutations, it is important to assess the interaction among mutations in relation to viral replication fitness and the possible impact on therapy [2,40-42]. For example, in contrast to the severe replication defect conferred by L74V mutation in the background of K65R [5,29], RT mutation A62V and S68G have been shown to improve replication capacity of virus when selected in the same genome that contain K65R mutation [7]. Other studies have demonstrated that the RT mutation M184V further decreases replication capacity of K65R viruses by decreasing the ability to incorporate natural dNTPs [32,40,43]. In the context of L74I selection, a recent survey of large database revealed that TAMs and M184V are the most commonly observed nucleoside analogue mutations (>25%) followed by L74V/I (11%) and K65R remain stable (3.3%) between 2003-2006 [1,3,5]. The significant linkage studies by Parikh *et al *(2006) had previously demonstrated that while TAMs are rarely observed in combination with K65R their association with L74V/I is more frequent [3,44]. Another study focusing on the selection parameters for L74V versus L74I mutations showed that the selection of the latter is more frequent under zidovudine and abacavir combination or under tenofovir with the presence of TAMs [27,45]. They further showed that K103N is also associated with L74I emergence in the absence of other NNRTI mutations (L100I, G190A and Y181C). In contrast, the selection of L74V is mainly associated with the use of didanosine. This study showed that the selection of L74V and L74I is controlled by two independent pathways and it is speculated that the resistance levels and replication capacity of viruses containing these mutations may be different. It is conceivable that the robust RCs of L74I viruses will have an implication in the selection and prevalence of mutant viruses with L74I mutation presumably with thymidine analogue mutations under specific combination of drugs. Our observations that L→I but not L→V change at RT codon 74 in the background of K65R leads to the generation of RT which is much more stable and enough for the enhanced viability of the virus (Figure 1 and Figure 4) is intriguing and needs to be addressed further. Specifically, the impact of emerging L74I in combination of other NRTI-selected mutations should be analyzed. Considering the low level selection of K65R mutation in treatment-experienced patients exposed to abacavir or didanosine, which also select L74V [46], and the observations that patients with K65R experienced significantly higher rates of virologic suppression than did those with L74V [26] requires further virological and biochemical investigation to understand the interactions among RT residues at codon 65 and 74 including impact of amino acid polymorphism.

## Conclusions

In summary, we demonstrated here that in contrast to L74V, the L74I mutation in the background of K65R results in a replication competent virus due to an increased processivity of RT. As both the double mutants are attenuated in comparison to WT and single point mutants (K65R, L74V and L74I) and RTs containing mutations K65R+L74V or K65R+L74I have decreased processivity, our results provide the explanation for the rarity of these double mutants in clinical settings. Our observations emphasize the significance of better understanding and identification of the novel amino acids of RT that are highly deleterious when mutated, in order to optimize drug regimens during virologic failure, design novel RT inhibitors, and analyze vaccine constructs targeted to specific CD8^+ ^T-cell responses against these targets.

## Abbreviations

PBM: peripheral blood mononuclear cells; RT: reverse transcriptase; HIV-1: human immunodeficiency virus type 1; dNTPs: deoxynucleotide triphosphates; PCR: polymerase chain reaction; TAMs: thymidine analogue mutations; NRTI: nucleoside reverse transcriptase inhibitors; NNRTI: non-nucleoside reverse transcriptase inhibitors

## Competing interests

The authors declare that they have no competing interests.

## Authors' contributions

All authors have made major contributions to this study. PLS was involved in overall conceptualization of the idea and performing processivity assays. HBC was involved in site directed mutagenesis, replication kinetic assays and preparing data for the manuscript. VN played an important role initially in the project for designing oligonucleotides for mutagenesis and sequencing, plasmid DNA preparation, transformation in bacteria and transfection in mammalian cells to prepare viruses. DR and CC were involved in analysis of data and suggesting experimental design for the study. All authors have read and approved the final manuscript.

## Authors' information

PLS is an Assistant Professor in the Department of Microbiology and Immunology and has more than 15 years of experience in the area of HIV-1 replication fitness. PLS has published key papers in this area that are cited in this manuscript. HBC is a post doctoral associate and is working in the area of HIV replication fitness since Jan. 2008. VN is currently a resident in Family medicine and has worked in the laboratory of PLS for three years. VN has published several papers with PLS in the area of HIV-1 replication fitness. DR is Professor of Medicine at Emory University and Chief of Infectious Diseases at Atlanta VA Medical Center. DR is involved in HIV clinical research since past 20 years. CC is Professor of Medicine at Harvard Medical School and has published several key papers in the area of HIV-1 replication fitness and RT processivity with PLS.
